# Endovascular Reconstruction of a Stenotic Persistent Primitive Trigeminal Artery Causing Hemodynamic Vertigo: A Case Report

**DOI:** 10.7759/cureus.114541

**Published:** 2026-08-14

**Authors:** Tejesh Jagannatham, Preethi Vijayasekar, Ansan Joseph, Sabarish Sekar, Karthik Kulanthaivelu

**Affiliations:** 1 Neuroimaging and Interventional Radiology, Sri Ramachandra Institute of Higher Education and Research, Chennai, IND

**Keywords:** cervical vestibular evoked myogenic potential, endovascular stenting, intracranial atherosclerotic stenosis, persistent primitive trigeminal artery, vertebrobasilar insufficiency

## Abstract

Atherosclerotic stenosis of a persistent primitive trigeminal artery is an exceptionally rare cause of posterior circulation hypoperfusion. A 48-year-old woman with hypertension and hyperlipidemia presented with recurrent, disabling rotational vertigo despite six weeks of aggressive medical therapy. Comprehensive neurological and otolaryngological evaluation, including a work-up to exclude common peripheral vestibular disorders, supported a vascular etiology for her persistent symptoms. Magnetic resonance angiography and digital subtraction angiography demonstrated a Saltzman type I persistent primitive trigeminal artery with severe ostial stenosis and coexisting 60%-70% stenosis of the ipsilateral cavernous internal carotid artery. The posterior circulation was predominantly dependent on the carotid-trigeminal axis because vertebrobasilar inflow was limited and the posterior communicating arteries were absent. Cervical vestibular evoked myogenic potential responses were absent bilaterally, supporting dysfunction of the sacculocollic pathway in the setting of suspected posterior circulation hypoperfusion. The persistent primitive trigeminal artery stenosis was treated first with sequential balloon angioplasty and deployment of a 2.0 mm × 8 mm zotarolimus-eluting balloon-expandable coronary stent, followed by angioplasty and self-expanding stent placement across the cavernous internal carotid artery stenosis. Final angiography demonstrated restoration of brisk flow through the carotid-trigeminal axis and basilar artery. At three months, the patient had near-complete resolution of vertigo, and bilateral cervical vestibular evoked myogenic potential responses were restored. This case illustrates that, following comprehensive clinical evaluation, exclusion of alternative causes of vertigo, and detailed assessment of collateral anatomy and procedural risk, individualized endovascular reconstruction may be considered in carefully selected patients with medically refractory symptoms and a hemodynamically dominant persistent primitive trigeminal artery.

## Introduction

The persistent primitive trigeminal artery is the most common persistent embryologic carotid-vertebrobasilar anastomosis and is identified in approximately 0.1%-0.6% of cerebral angiographic examinations [1,2]. During normal embryogenesis, transient carotid-vertebrobasilar channels regress as the posterior circulation develops. Persistence into adulthood may alter posterior circulation hemodynamics and influence the presentation and treatment of cerebrovascular disease.

Vertigo is a common clinical symptom with a broad differential diagnosis, most frequently resulting from peripheral vestibular disorders such as benign paroxysmal positional vertigo and Ménière disease. However, vertebrobasilar insufficiency should be considered in patients with recurrent disabling vertigo, vascular risk factors, and imaging findings suggestive of posterior circulation hypoperfusion.

In the Saltzman type I configuration, the persistent primitive trigeminal artery supplies the distal basilar artery, while the vertebral arteries may be hypoplastic and the posterior communicating arteries absent [1,2]. Consequently, the internal carotid artery-persistent primitive trigeminal artery axis can become the dominant inflow route to the posterior circulation. Although persistent primitive trigeminal arteries are more commonly discussed in association with aneurysms and carotid-cavernous fistulas [3], symptomatic atherosclerotic stenosis is rare, and reports of endovascular treatment are limited [4].

We describe a patient with medically refractory hemodynamic vertigo associated with severe stenosis of a hemodynamically dominant persistent primitive trigeminal artery and coexisting cavernous internal carotid artery stenosis, successfully managed with individualized endovascular reconstruction.

## Case presentation

Clinical presentation

A 48-year-old woman with hypertension and hyperlipidemia presented with a six-month history of recurrent rotational vertigo. Episodes lasted three to eight minutes, occurred two to four times per week, and were occasionally triggered by positional change. They were associated with nausea and a sensation of imbalance, without hearing loss, tinnitus, diplopia, dysarthria, limb weakness, sensory loss, or loss of consciousness. Neurologic examination showed mild truncal instability without focal motor, sensory, cerebellar, or cranial nerve deficits. Given the recurrent disabling nature of her symptoms, the patient underwent comprehensive neurological and otolaryngological evaluation. Common peripheral vestibular disorders, including benign paroxysmal positional vertigo and Ménière disease, were excluded following comprehensive otolaryngological assessment. She was initially managed conservatively with aggressive medical therapy, including dual antiplatelet therapy, statin therapy, antihypertensive optimization, and symptomatic vestibular-suppressant medication. Despite six weeks of optimal medical management, her symptoms remained frequent and disabling, prompting further vascular evaluation.

Imaging and physiologic assessment

**Figure 1:**
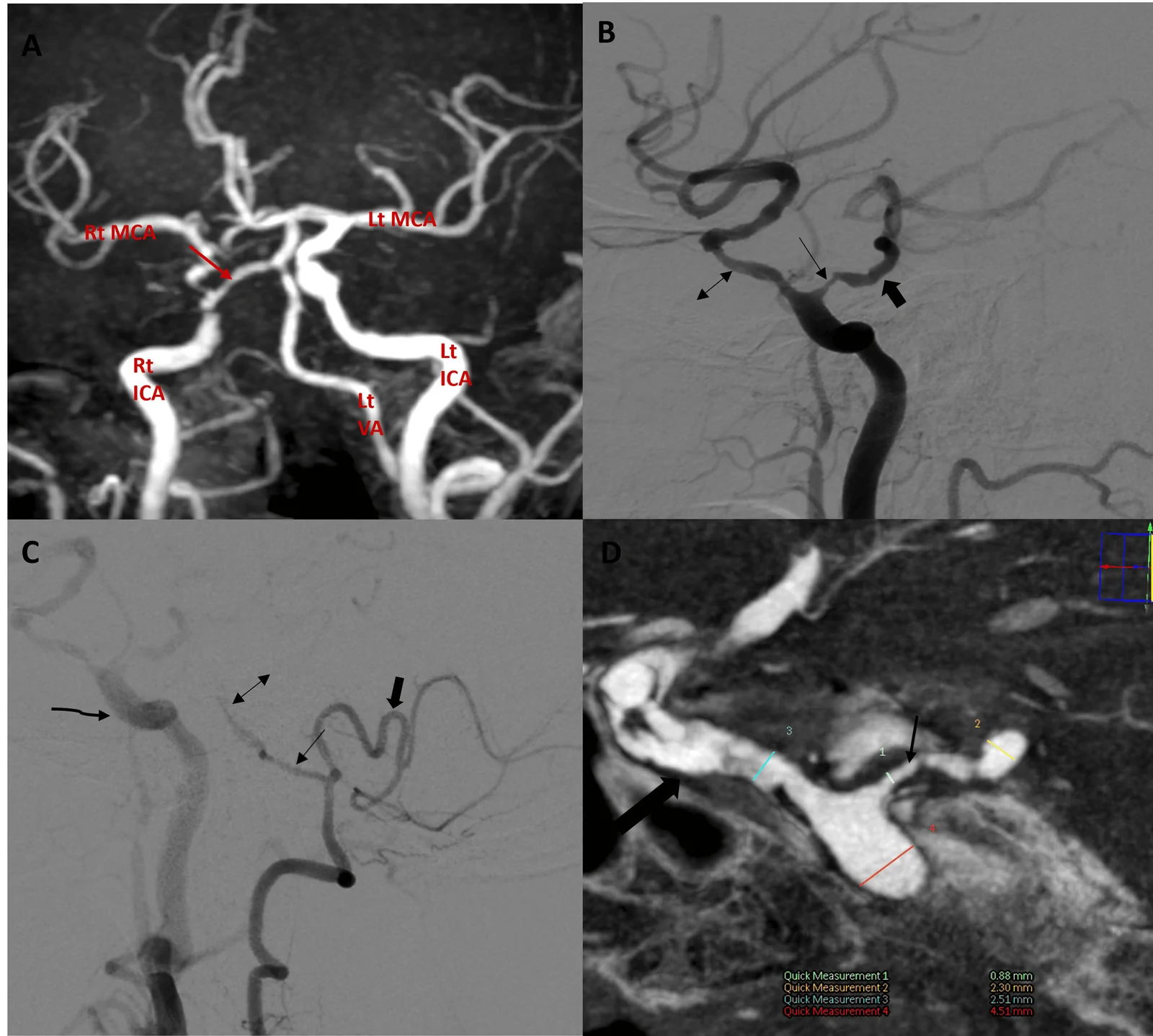
Preprocedural vascular imaging (A) Time-of-flight magnetic resonance angiography demonstrates a Saltzman type I persistent primitive trigeminal artery (arrow) with ostial stenosis and coexisting right cavernous internal carotid artery stenosis. (B) Lateral right internal carotid angiography demonstrates severe short-segment ostial stenosis (long arrow) of the persistent primitive trigeminal artery (block arrow) and long-segment stenosis of the cavernous internal carotid artery (double-headed arrow). (C) Lateral right vertebral angiography demonstrates a diminutive vertebral artery (arrow) with minimal contribution to the basilar artery (double-headed arrow). The curved arrow indicates the right internal carotid artery, and the block arrow indicates the posterior inferior cerebellar artery. (D) Cone-beam CT angiography demonstrates irregular luminal narrowing of the right cavernous internal carotid artery (block arrow) and severe ostial-proximal stenosis of the persistent primitive trigeminal artery (arrow). Measurements confirm severe stenosis according to the Warfarin-Aspirin Symptomatic Intracranial Disease (WASID) method [5]. ICA: Internal Carotid Artery, MCA: Middle Cerebral Artery, Rt: Right, Lt: Left, VA: Vertebral Artery.

Brain MRI, including diffusion-weighted imaging, showed no acute infarction. Time-of-flight magnetic resonance angiography demonstrated a right persistent primitive trigeminal artery arising from the cavernous internal carotid artery, with severe short-segment ostial stenosis. Digital subtraction angiography confirmed the severe ostial stenosis, quantified using the Warfarin-Aspirin Symptomatic Intracranial Disease (WASID) method [5], and demonstrated a Saltzman type I configuration in which the basilar artery and both posterior cerebral arteries were predominantly supplied through the persistent primitive trigeminal artery. The right vertebral artery was diminutive, measuring less than 2 mm, and neither posterior communicating artery was visualized. An additional 60%-70% atherosclerotic stenosis was identified in the cavernous segment of the internal carotid artery distal to the origin of the persistent primitive trigeminal artery (Figure 1).

As part of the evaluation for recurrent vertigo, cervical vestibular evoked myogenic potential (cVEMP) testing demonstrated absent bilateral responses, indicating dysfunction of the sacculocollic pathway. Following exclusion of common peripheral vestibular disorders and structural intracranial pathology, the angiographic findings, together with the abnormal cVEMP responses, supported posterior circulation hypoperfusion as the most likely mechanism of the patient's symptoms [6,7].

Medical management

The patient was treated with aspirin 75 mg daily, clopidogrel 75 mg daily, atorvastatin 40 mg daily, and optimization of antihypertensive therapy. Symptomatic treatment with vestibular-suppressant medication was also administered during the period of conservative management. Vestibular rehabilitation therapy was not pursued because comprehensive neurological and otolaryngological evaluation favored a hemodynamic vascular etiology rather than a peripheral vestibular disorder. Despite six weeks of aggressive medical management, the disabling episodes continued without meaningful reduction in frequency or severity.

Endovascular procedure

**Figure 2:**
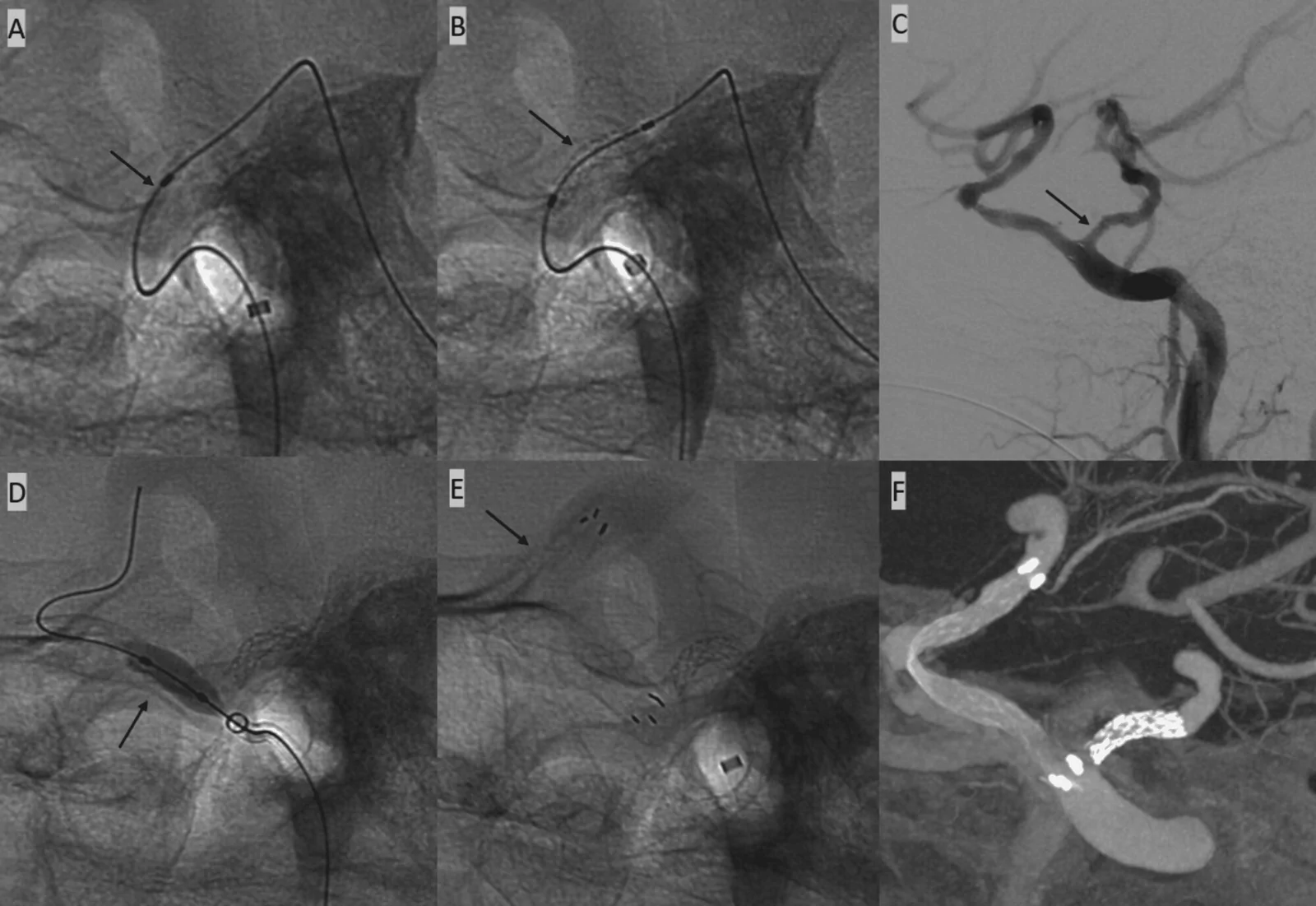
Endovascular reconstruction (A) Balloon angioplasty of the persistent primitive trigeminal artery stenosis using a 1.5 mm × 8 mm balloon (arrow). (B) Deployment of a 2.0 mm × 8 mm balloon-expandable drug-eluting stent across the persistent primitive trigeminal artery stenosis (arrow). (C) Post-stenting angiography demonstrates marked luminal gain at the persistent primitive trigeminal artery origin (arrow). (D) Balloon angioplasty of the cavernous internal carotid artery stenosis (arrow). (E) Deployment of the Enterprise 2 Vascular Reconstruction Device (Cerenovus, Irvine, CA, USA) across the cavernous internal carotid artery stenosis (arrow). (F) Maximum-intensity-projection image from rotational angiography demonstrates the persistent primitive trigeminal artery and cavernous internal carotid artery stents in situ, with preserved flow.

After written informed consent, the procedure was performed under general anesthesia through right common femoral artery access using an 8-French sheath. Systemic heparinization was used to maintain an activated clotting time of 250-300 seconds. An 8-French Cerebase guide sheath (Cerenovus, Irvine, CA, USA) was positioned in the distal cervical internal carotid artery, and a 5-French AXS Catalyst 5 distal access catheter (Stryker Neurovascular, Fremont, CA, USA) was advanced into the petrous internal carotid artery to provide triaxial support. A Synchro2 0.014-inch microwire (Stryker Neurovascular) was navigated across the acutely angulated persistent primitive trigeminal artery origin into the basilar artery. Sequential predilation was performed with 1.0 mm × 6 mm and 1.5 mm × 8 mm coronary balloons. A 2.0 mm × 8 mm Resolute Onyx zotarolimus-eluting balloon-expandable coronary stent (Medtronic, Santa Rosa, CA, USA) was then deployed across the persistent primitive trigeminal artery ostium at 10 atm, producing substantial luminal gain. A balloon-expandable drug-eluting stent was selected for precise ostial deployment and adequate radial force, with the additional potential advantage of reducing restenosis in this small-caliber vessel [8]. The cavernous internal carotid artery stenosis was treated after reconstruction of the persistent primitive trigeminal artery. Predilation was performed with a 3.0 mm × 8 mm coronary balloon, followed by deployment of a 4.0 mm × 30 mm Enterprise 2 self-expanding intracranial stent (Cerenovus). The persistent primitive trigeminal artery was reconstructed first to secure the dominant posterior circulation inflow and preserve direct access to its ostium. The cavernous internal carotid artery stenosis was subsequently treated because of its severity and the perceived risk that progression, flow stasis, or thrombosis could compromise the reconstructed carotid-trigeminal axis. Final angiography demonstrated brisk antegrade flow through the internal carotid artery and persistent primitive trigeminal artery, with robust opacification of the basilar artery and posterior cerebral arteries (Figure 2).

Clinical outcome

The patient remained neurologically intact after the procedure and was discharged on postprocedural day five while continuing dual antiplatelet therapy. At the three-month follow-up, she reported one mild episode of vertigo during the interval, compared with eight to 12 episodes per month before treatment. Repeat cVEMP testing demonstrated restoration of bilateral responses.

## Discussion

This case demonstrates the clinical importance of recognizing posterior circulation dependence on a persistent primitive trigeminal artery. In a Saltzman type I configuration with a diminutive right vertebral artery, limited vertebrobasilar inflow, and absent posterior communicating arteries, stenosis involving the dominant carotid-trigeminal circulation may significantly compromise posterior circulation perfusion [2].

Vertigo is a common clinical complaint with a broad differential diagnosis, most frequently arising from peripheral vestibular disorders such as benign paroxysmal positional vertigo, Ménière disease, vestibular neuritis, and vestibular migraine. In contrast, vertebrobasilar insufficiency is an uncommon but important central cause that should be considered in patients with recurrent disabling vertigo, vascular risk factors, and imaging findings suggestive of posterior circulation hypoperfusion. Consequently, careful clinical evaluation, including neurological and otolaryngological assessment, is essential before attributing symptoms to a vascular etiology and considering invasive treatment. In our patient, common peripheral vestibular disorders were excluded following comprehensive otolaryngological evaluation, and the diagnosis of hemodynamic vertebrobasilar insufficiency was supported by the unique vascular anatomy, abnormal cVEMP findings, and failure of optimal conservative medical therapy.

Vertigo due to vertebrobasilar insufficiency can be difficult to establish when diffusion-weighted imaging is negative and focal neurological deficits are absent. The labyrinthine circulation is particularly vulnerable because the internal auditory artery is usually an end artery arising from the anterior inferior cerebellar artery or, less often, directly from the basilar artery. Cervical vestibular evoked myogenic potential testing evaluates the saccule, inferior vestibular nerve, vestibular nuclei, and vestibulocollic pathways [7]. Although an absent response is not specific for vascular insufficiency, the bilateral abnormality before treatment and recovery after revascularization provided supportive physiological correlation with the clinical and angiographic findings [6,7].

Endovascular treatment of intracranial atherosclerotic disease must be considered cautiously because randomized trials have shown no additional benefit and substantial early procedural risk when stenting is broadly applied [9,10]. More favorable outcomes in carefully selected, delayed, on-label cohorts emphasize the importance of strict patient selection, operator experience, and procedural timing [11,12]. Current evidence supports aggressive medical management, including dual antiplatelet therapy, intensive lipid-lowering therapy, and optimization of vascular risk factors, as the initial treatment strategy for symptomatic intracranial atherosclerotic disease [9,10]. The anatomy in the present case, however, was unusual: the stenotic persistent primitive trigeminal artery represented the principal conduit to the posterior circulation, symptoms remained frequent and disabling despite optimal medical therapy, and collateral circulation was markedly limited. The procedure was therefore undertaken as an individualized rescue strategy rather than as routine treatment of intracranial atherosclerotic stenosis.

The intervention posed several technical challenges, including the small caliber and acute takeoff of the persistent primitive trigeminal artery, limited collateral support, and the potentially catastrophic consequence of vessel occlusion. A triaxial access system improved stability, while sequential low-profile balloon angioplasty reduced the force required to cross and expand the lesion. The balloon-expandable drug-eluting stent permitted accurate ostial placement and provided radial support in a vessel approximately 2 mm in diameter. Data from symptomatic intracranial stenosis suggest that drug-eluting stents may reduce in-stent restenosis compared with bare-metal stents in selected patients, although the applicability of those data to persistent primitive trigeminal artery stenosis is uncertain [8].

The treatment sequence also required careful procedural planning. Reconstruction of the persistent primitive trigeminal artery was performed first to secure the dominant posterior circulation inflow and preserve direct access to its ostium. Subsequently, the coexisting cavernous internal carotid artery stenosis was treated based on individualized procedural considerations, including the perceived risks of stenosis progression, flow stasis, thrombosis, and potential compromise of the persistent primitive trigeminal artery origin. Unlike classic tandem lesions encountered in acute ischemic stroke, the vascular anatomy in the present case was unique; therefore, treatment decisions were individualized according to the patient's specific cerebrovascular anatomy rather than established tandem-lesion strategies.

Published experience with atherosclerotic stenosis of a persistent primitive trigeminal artery remains limited to isolated reports [4]. Accordingly, long-term imaging follow-up is required to assess stent patency and restenosis [12], and conclusions regarding safety or generalizability cannot be drawn from a single case. In addition, although clinical improvement followed reconstruction of both lesions, the independent contribution of cavernous internal carotid artery stenting to the patient's symptomatic improvement cannot be established and should therefore be interpreted with caution.

## Conclusions

A hemodynamically dominant persistent primitive trigeminal artery can become a critical posterior circulation conduit, and severe stenosis may present with recurrent vertigo even without established infarction. Following comprehensive clinical evaluation and exclusion of common peripheral causes of vertigo, individualized endovascular reconstruction may be considered in carefully selected patients with disabling symptoms despite aggressive medical therapy, with treatment of associated vascular lesions when clinically appropriate. In this patient, the approach was associated with restoration of posterior circulation flow, marked clinical improvement, and recovery of bilateral cVEMP responses. Detailed evaluation of collateral anatomy, cautious patient selection, and meticulous protection of the persistent primitive trigeminal artery origin are essential, while long-term angiographic surveillance remains necessary. Given the rarity of this condition, further studies are required to better define the role of endovascular treatment and its long-term outcomes.
